# Testing of a femtosecond pulse laser in outer space

**DOI:** 10.1038/srep05134

**Published:** 2014-05-30

**Authors:** Joohyung Lee, Keunwoo Lee, Yoon-Soo Jang, Heesuk Jang, Seongheum Han, Sang-Hyun Lee, Kyung-In Kang, Chul-Woo Lim, Young-Jin Kim, Seung-Woo Kim

**Affiliations:** 1 Department of Mechanical Engineering, Korea Advanced Institute of Science and Technology (KAIST), Science Town, Daejeon, 305-701, South Korea; 2 Satellite Technology Research Center (SaTReC), Korea Advanced Institute of Science and Technology (KAIST), Science Town, Daejeon, 305-701, South Korea; 3 These authors contributed equally to this work.; 4 Current address: Center for Space Optics, Korea Research Institute of Standards and Science (KRISS), Science Town, Daejeon, 305-340, South Korea

## Abstract

We report a test operation of an Er-doped fibre femtosecond laser which was conducted for the first time in outer space. The fibre-based ultrashort pulse laser payload was designed to meet space-use requirements, undergone through ground qualification tests and finally launched into a low-earth orbit early in 2013. Test results obtained during a one-year mission lifetime confirmed stable mode-locking all the way through although the radiation induced attenuation (RIA) in the Er-doped gain fibre caused an 8.6% reduction in the output power. This successful test operation would help facilitate diverse scientific and technological applications of femtosecond lasers in space and earth atmosphere in the near future.

With advancement during the last few decades on earth^1^, the frequency comb of mode-locked femtosecond pulse lasers is now intended to play important roles for various space missions anticipated in the near future^2^,^3^. In this regard, a prerequisite is securing femtosecond laser sources capable of enduring high-g launching vibration, space thermal-vacuum environment and high energy radiation. Here, we report a test operation of a fibre-based femtosecond oscillator conducted in outer space over a year. The femtosecond laser payload was carefully designed to meet space-use requirements, undergone through ground qualification tests and finally launched into an orbit in January 30, 2013. Throughout a one-year mission lifetime, mode-locked operation was confirmed stable with an 8.6% reduction in the average output power due to the radiation-induced attenuation in the Er-doped gain fibre. This successful operation would help facilitate diverse scientific and technological applications of femtosecond lasers in space and earth atmosphere.

The frequency comb of mode-locked femtosecond pulse lasers has led to remarkable advances in many fields; high resolution spectroscopy^4^,^5^, broad band calibration of astronomical spectrographs^6^,^7^,^8^, time/frequency transfer over long distances^9^,^10^,^11^, absolute laser ranging^12^,^13^,^14^,^15^, precision strain sensing^16^, and inter-comparison of atomic clocks^17^,^18^. Now the frequency comb is anticipated to be applied directly to space missions in the near future; examples include experimental validation of the theory of general relativity^19^,^20^, high precision mapping of the geo-potential^21^,^22^ and large synthetic aperture imaging^23^,^24^,^25^. The frequency comb employed for space missions should be capable of surviving launching acceleration^26^,^27^ and also sustaining harsh thermal-vacuum environment of outer space^28^. More importantly, the frequency comb should be robust enough, optically and electrically, to endure high energy space radiation^29^,^30^. In this respect, fibre-based femtosecond lasers are a preferential choice due to their high alignment stability, small footprint, light weight and power conversion efficiency, but their immunity to space radiation has not carefully been verified for actual space applications requiring a long mission lifetime.

## Results and Discussion

### Femtosecond pulse laser for space operation

**Figure 1:**
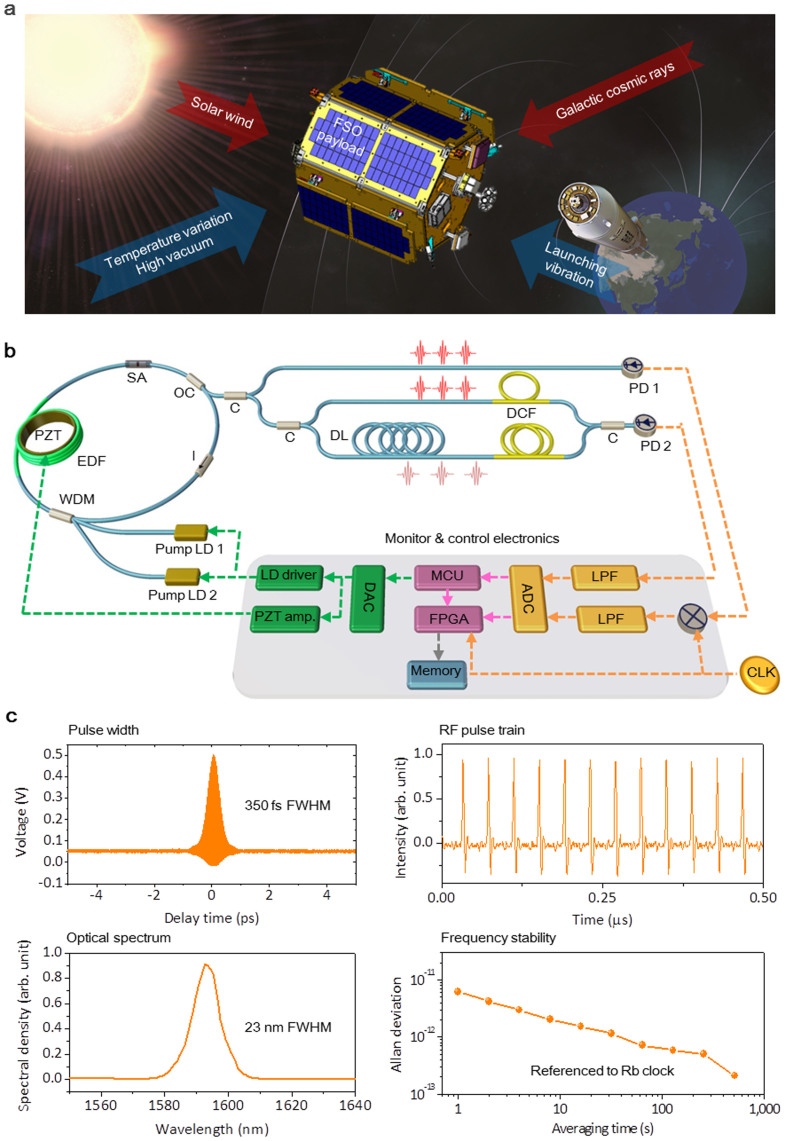
Design of an all-fibre femtosecond laser oscillator (FSO) for space operation. (a), Artistic image presenting harsh environment factors in outer space (drawn by Y.-S. J. and S.-H.L.); 10-g launching acceleration, 60-K temperature variation and 4.80-krad total ionization dose over a year. (b), Overall hardware configuration of FSO. EDF: erbium-doped fibre, PZT: piezo-electric actuator, WDM: wavelength division multiplexer, I: isolator, OC: output coupler, C: coupler, DL: delay line, DCF: dispersion compensating fibre, PD: photo detector, LD: laser diode, DAC: digital-to-analog converter, ADC: analog-to-digital converter, MCU: micro-controller unit, FPGA: field programmable gate array, LPF: low-pass filer and CLK: clock. (c), Ground test results on the pulse duration, RF pulse train, optical spectrum, and frequency instability of the repetition rate.

In our investigation, a fibre femtosecond laser was operated in space as an attempt ever made for the first time to our knowledge. The femtosecond laser, named FSO in short, was carefully designed so as to meet space-use requirements, undergone through ground qualifications and finally launched into an orbit. As illustrated in Fig. 1, the hardware of FSO was basically comprised of optical and electrical components constituting an all-fibre ring-type oscillator. An Er-doped fibre was used as the gain medium. Mode-locking was activated using a saturable absorber (SA) of transmission type selected to be directly inserted within the oscillator without use of bulk optics. Pulses were generated in a soliton mode with a net dispersion of −1000 fs^2^ which was precisely managed within a 0.1% error by addition of a single-mode fibre within the oscillator loop^31^. Two identical pump laser diodes (LDs) of a 980 nm wavelength were installed; one as the main pump for normal operation and the other as a backup in case the main pump breaks down during the mission lifetime of a year. The pulse repetition rate was made actively be regulated by varying the oscillator cavity length using a ring-type piezoelectric actuator (PZT) where the Er-doped gain fibre was glued around. After assembly, laboratory tests were conducted to confirm that the oscillator emitted ultrashort pulses of 350 fs duration at a 25 MHz repetition rate. The average output power was measured to be ~14 mW for a pump power of 600 mW. The output spectral bandwidth was 23 nm in full-width-half-maximum (FWHM) centred at a 1590 nm wavelength. The fractional instability of the phase-locked pulse repetition rate was measured to be 1 × 10^−12^ at 10 s averaging using a frequency counter referenced to the Rb atomic clock.

For remote operation in orbit, FSO was supplemented with an electronic control board specially built to accommodate a micro-controller unit (MCU) which was pre-programmed to execute the operation scenario upon receiving the command from the ground station. On board was also a field-programmable gate array (FPGA) dedicated to monitor the operation state of FSO with capabilities of high speed data acquisition, processing and storage at a 200 MHz sampling rate. The MCU and FPGA were wired to monitor the average output power and pulse repetition rate through an InGaAs photodiode (PD1) of a 6 GHz bandwidth. Besides, an unequal-path fibre interferometer was attached to the output port of FSO, which was intended to estimate the pulse duration and optical spectrum by capturing the 1^st^-order autocorrelation of generated pulses. All the performance data measured in real time was stored in an on-board memory provisionally and later sent down to the ground station when requested. More details of FSO's hardware and data processing are given in Supplementary Information.

### Design details for space operation

**Figure 2:**
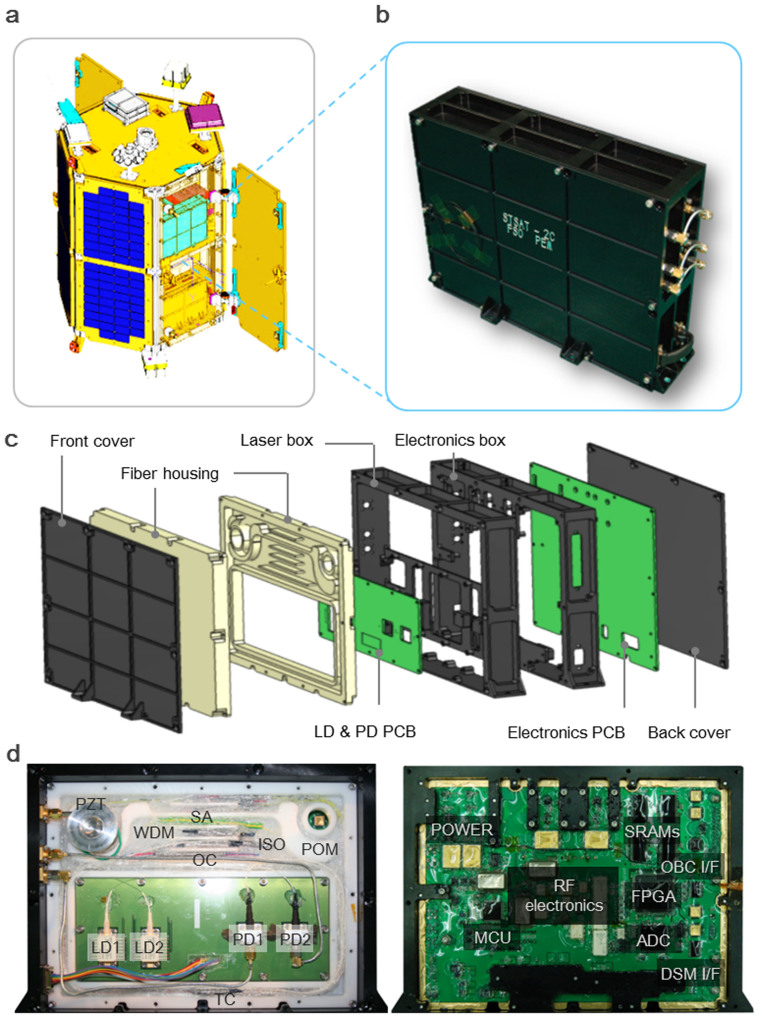
FSO payload. (a), STSAT-2C scientific satellite (drawn by S.-H. Lee). (b), Assembled FSO payload. (c), Expanded view of FSO payload. PCB: printed circuit board, LD: laser diode, PD: photodetector. (d), Open views of the optical compartment (left) and electronic compartment (right).

FSO was loaded on a 100 kg class scientific satellite (STSAT-2C) which was carried by Naro-3 carrier rocket (KSLV-1) into an elliptical low Earth orbit (perigee 292 km, apogee 1511 km). As shown in Fig. 2, FSO was assembled within an aluminium (Al6061-T6) container which occupies a 3.3 × 10^−3^ m^3^ working volume and weighs 2.5 kg. The FSO payload was designed to maximize the protection capability against vibration and heat within the given constraints on its volume, footprint and weight^32^,^33^. The designed structure (Figs. 2a to 2c) was comprised of two separate compartments – one for optics and the other for electronics – with a thermal barrier in between to reduce the heat crosstalk between optical components and electrical devices. Vibration and heat transfer analysis was performed by using the Finite Element Method (FEM). (More details are given in Supplementary Information). In order to endure the estimated launching acceleration of ~98 m/s^2^ (~10 g) and temperature variation of −20 to 50°C in the orbit, special care was taken to secure all the fibres around thermally-stable spools with pre-stress to minimize time-dependent polarization change. And, the electrical components including laser diodes were hermetically sealed on a space-qualified FP7 polymer board using low-volatility space-grade silicon for higher immunity to vibration. In addition, the whole optical assembly was packaged into a polyoxymethylene box of low thermal conductivity for protection from temperature fluctuation without active temperature stabilization. Heat generated from the electric power consumption of 20 W inside FSO was made dissipated in vacuum environment by conduction through the satellite's main frame.

### Ground-level environmental tests

**Figure 3:**
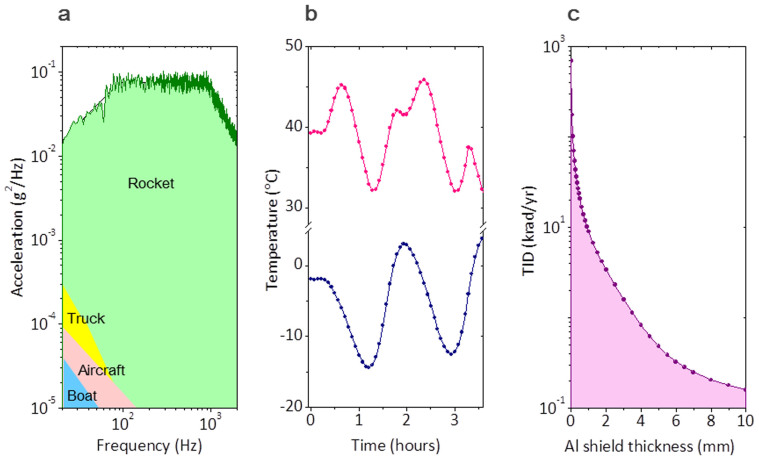
Environmental test conditions. (a), Launching acceleration spectrum. (b), Temperature variation in orbit. (c), Space radiation level for given Al shield thickness.

During the development stage of FSO, a qualification model was prepared for ground-level tests to validate its endurance to three space environment factors; vibration, thermal-vacuum and gamma-ray radiation in accordance with the NASA's standard guidelines^28^. Test specifications were drawn in terms of vibration, temperature and space radiation as shown in Fig. 3. First, the vibration test criterion was given in a form of trapezoidal acceleration spectrum (Fig. 3a), which was deduced from the launching data of the Naro-3 rocket (KSLV-1). The given spectrum was equivalent to a total integrated acceleration of 139 m/s^2^ (14.2 g), which was far above most military vibration requirements for boats, aircrafts and trucks^34^. Second, the temperature test requirement (Fig. 3b) was prepared in response to two extreme cases (red line for full sun and blue line for eclipse period), which the FSO payload would encounter in the given orbit of the STSAT-2C satellite. Third, the space radiation level (Fig. 3c) was estimated in consideration of high energy electrons, protons and heavy ions trapped from galactic cosmic rays and solar winds into Van Allen radiation belts^35^,^36^. The required test level was quantified in terms of the total ionization dose (TID) per a one-year mission lifetime as a function of the aluminium (Al) shield thickness of the FSO payload.

**Figure 4:**
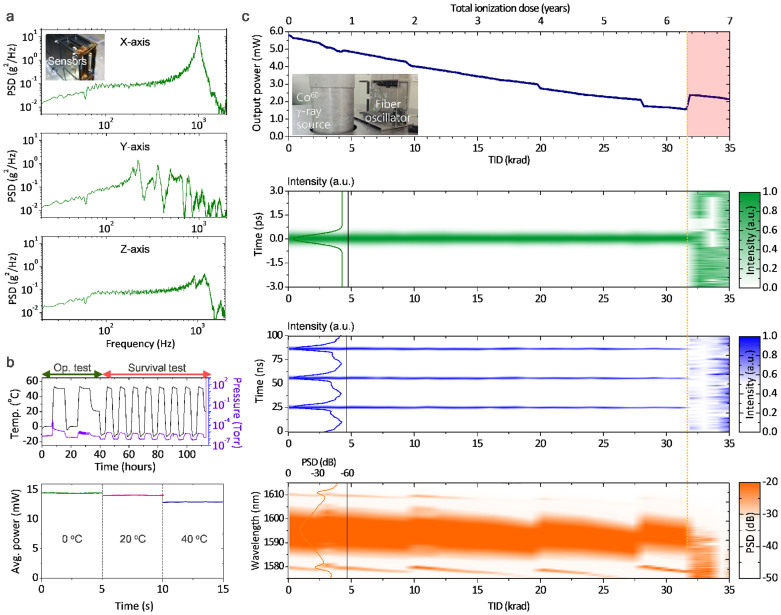
Space qualification tests on the ground. (a), Launching vibration test results. Total integrated acceleration level was set at 14.2 g. (b), Thermal vacuum test results for a sequence of two operational and eight survival test cycles. (c), Space radiation test results. Accumulative space radiation was simulated using a ^60^Co gamma-ray radiation source. A total ionization dose (TID) of 147 krad was exposed while monitoring the pulse duration, RF pulse train, and optical spectrum in real time.

Ground-level space environmental tests were faithfully conducted prior to launching. First, an integrated acceleration of 139 m/s^2^ (14.2 g) was applied along the x-, y- and z- direction, respectively, using an electro-magnetic exciter generating a random vibration for 60 seconds. The measured power spectral density (PSD) of each directional vibration amplitude was found in good agreement with the theoretically prediction made in the design stage using finite element analysis (Fig. 4a). Neither structural nor functional damage was observed in the qualification model after the vibration test, indicating that FSO is mechanically fit to sustain the maximum launching acceleration of 98 m/s^2^ (10 g). Second, thermal-vacuum test was performed over −10 to 50°C under 10^−3^ ~ 10^−4^ Pa (10^−5^ ~ 10^−6^ torr) pressure level through two operational test cycles and eight survival test cycles with a 90 minute dwell period at each temperature peak and valley (Fig. 4b). Operational test results confirmed mode-locking was active when temperature was kept in the range of 0 to 45°C. Outside the temperature region, Q-switch mode-locked pulses or multi-pulsing were observed due to too low or excessively high intra-cavity circulation power. The output power during the operation cycles showed a decreasing tendency with increasing temperature. After all the survival cycles were complete, FSO returned to normal operation in the room temperature with no sign of thermal damage.

### Ground-level space radiation test

Last but not least, radiation test was conducted by exposing FSO to ^60^Co gamma-ray radiation (1.17 and 1.33 MeV). An accumulated amount of 147 krad(H_2_O) TID (hereafter, ‘krad' indicates ‘krad(H_2_O)' as specified in Ref [37]) was deposited over 17 hours with an 8.73 krad/hour dose rate while temperature was kept constant at ~15°C. The dose rate was selected in consideration of the radiation testing conditions of fibre optic components reported in Ref [29]. Test results (Fig. 4c) revealed that mode-locking began to break down when the gamma-ray TID reached 31.6 krad, which was detected by obvious instabilities observed in the pulse duration, pulse train and optical spectrum monitored simultaneously. The threshold TID value implies that FSO is capable of sustaining a 6.58 year normal operation in its orbit, of which annual TID to FSO is estimated to be 4.80 krad using the Space Environment Information System (SPENVIS) provided by European Space Agency (ESA)^38^. The output power gradually reduced to 30% of its original value until the mode-locking failure, which was attributed to the radiation induced attenuation (RIA) of the Er-doped gain fibre (LIEKKI^TM^ Er80-4/125, nLIGHT Co.) manufactured without special treatments for radiation resistance. Also monitored during the gamma-ray exposure were several sluggish shifts of the optical spectrum toward shorter wavelengths, each ending up with abrupt restoration to its original profile. Other components, i.e., the saturable absorber, photodetectors and pump laser diodes were tested individually and verified much more robust to RIA compared to the Er-doped gain fibre. (More details are given in Supplementary Information).

### FSO operation in outer space

**Figure 5:**
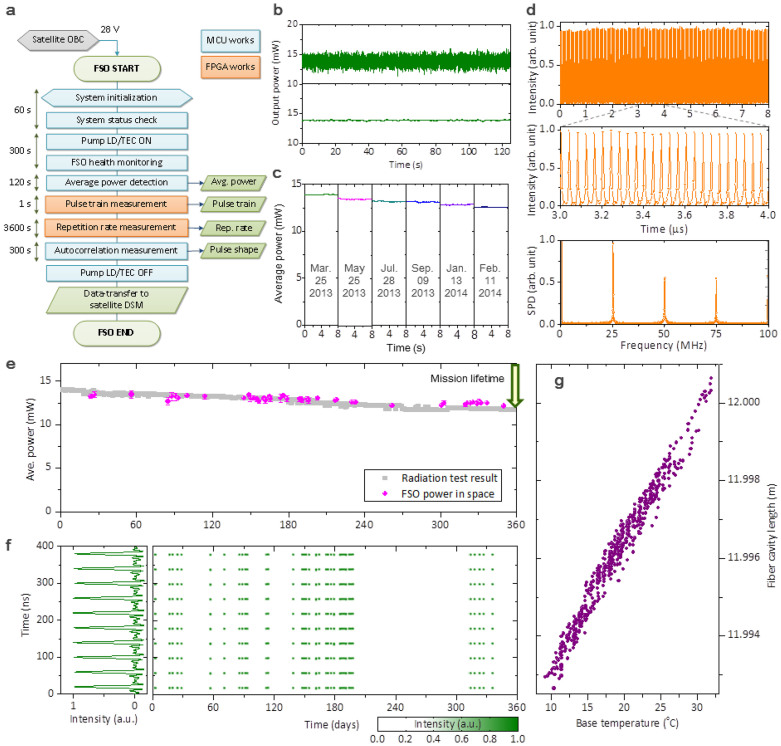
FSO operation results in outer space over a year. (a), FSO operation scenario in space. (b), Short-term stability of the output power monitored at 3 kHz (upper) and 1 Hz (lower) update rates. (c), Long-term variation of the output power due to RIA over a year. (d), Measured RF pulse train in the time domain and its RF spectrum. (e), Comparative plot of the measured output power over 12 months with the ground test result. (f), Long-term variation of the RF pulse train in the time domain over an year. (g), Length variation of the FSO cavity with temperature change.

Actual operation of FSO in space was initiated from early in 2013 by activating an operation scenario pre-programed on board (Fig. 5a) and ensuing performance data were collected in the ground station located on the KAIST campus, Daejeon, South Korea. The average output power observed over the last 12 months showed no noticeable systematic instability; its short-term fluctuation measured for 120 seconds was 3.5% and 0.42% (standard deviation) for a sampling rate of 3 kHz and 1 Hz, respectively (Fig. 5b). The power output exhibited a long-term tendency of gradual decrease due to the RIA in the Er-doped gain fibre, with a total reduction being 8.1% over the lifetime of a year (Fig. 5c), which is in good agreement with the gamma-ray radiation test performed on the ground before launching (Fig. 5e). The RF pulse train was also observed stable with a ~1% rms pulse peak intensity variation and a 1 × 10^−8^ stability of the repetition rate referenced to an on-board crystal clock (Fig. 5d). Direct observation of the pulse duration and optical spectrum in space by monitoring the 1^st^ order autocorrelation of generated pulses was not made. The reason was that active temperature control to establish a stable environment for the unbalanced interferometer was not permitted due to the electric power consumption limitation (20 W) allocated from the STSAT-2C mother satellite for FSO payload operation. Nevertheless, comparing the pulse train measured in space (Fig. 5f) with the ground qualification test results (Fig. 4c) concludes that FSO has maintained stable mode-locked operation without unwanted Q-switched mode-locking or multi-pulsing when temperature was within 10 to 35°C (Fig. 5g).

## Conclusions

To conclude, the Er-doped fibre femtosecond laser oscillator (FSO) which we designed for space test has completed a successful one-year operation. Test results confirmed stable mode-locking all the way through although the radiation induced attenuation (RIA) in the Er-doped gain fibre caused an 8.6% reduction in the output power. The expected lifetime in space was estimated to be 6.58 years for the current design but it would be extended to 65 years simply by increasing the aluminium shield thickness to 5 mm from the current value of 1.6 mm, or to a more extensive period by adopting radiation hardened Er-doped fibres which will be available in the near future^39^,^40^. This successful space operation of FSO would help facilitate diverse scientific and technological applications of the frequency comb of femtosecond lasers in space and Earth atmosphere in near future space missions.

## Author Contributions

The project was planned and overseen by S.-W.K., Y.-J.K. and S.-H.L. Payload assembly, ground qualifications and space tests were conducted by J.L., K.L., Y.-S.J., H.J., S.H., S.-H.L. and Y.-J.K. Satellite operation was performed by S.-H.L., K.-I.K., and C.-W.I. All authors contributed to the manuscript preparation.

## Supplementary Material

Supplementary Information Supplementary Information

## References

[b1] Diddams S. A. The evolving optical frequency comb. J. Opt. Soc. Am. B 27, B51–B62 (2010).

[b2] Kim S.-W. Combs rule. Nature Photon. 3, 313–314 (2009).

[b3] Newbury N. R. Searching for applications with a fine-tooth comb. Nature Photon. 5, 186–188 (2011).

[b4] Hänsch T. W. Nobel Lecture: Passion for precision. Rev. Mod. Phys. 78, 1297–1309 (2006).

[b5] Hall J. L. Nobel Lecture: Defining and measuring optical frequencies. Rev. Mod. Phys. 78, 1279–1295 (2006). 10.1002/cphc.200600457 17086589

[b6] Steinmetz T. *et al.* Laser frequency combs for astronomical observations. Science 321, 1335–1337 (2008). 18772434 10.1126/science.1161030

[b7] Li C.-H. *et al.* A laser frequency comb that enables radial velocity measurements with a precision of 1 cm s^−1^. Nature 452, 610–612 (2008). 18385734 10.1038/nature06854

[b8] Wilken T. *et al.* A spectrograph for exoplanet observations at the centimetre-per-second level. Nature 485, 611–614 (2012). 22660320 10.1038/nature11092

[b9] Fortier T. M. *et al.* Generation of ultrastable microwaves via optical frequency division. Nature Photon. 5, 425–429 (2011).

[b10] Predehl K. *et al.* A 920-kilometer optical fiber link for frequency metrology at the 19th decimal place. Science 336, 441–444 (2012). 22539714 10.1126/science.1218442

[b11] Giorgetta F. R. *et al.* Optical two-way time and frequency transfer over free space. Nature Photon. 7, 434–438 (2013).

[b12] Minoshima K. & Matsumoto H. High-accuracy measurement of 240-m distance in an optical tunnel by use of a compact femtosecond laser. Appl. Opt. 39, 5512–5517 (2000). 18354548 10.1364/ao.39.005512

[b13] Ye J. Absolute measurement of long, arbitrary distance to less than an optical fringe. Opt. Lett. 29, 1153–1155 (2004). 15182016 10.1364/ol.29.001153

[b14] Coddington I., Swann W. C., Nenadovic L. & Newbury N. R. Rapid and precise absolute distance measurements at long range. Nature Photon. 3, 351–356 (2009).

[b15] Lee J., Kim Y.-J., Lee K., Lee S. & Kim S.-W. Time-of-flight measurement with femtosecond light pulses. Nature Photon. 4, 716–720 (2010).

[b16] Gagliardi G., Salza M., Avino S., Ferraro P. & De Natale P. Probing the ultimate limit of fiber-optic strain sensing. Science 330, 1081–1084 (2010). 21030606 10.1126/science.1195818

[b17] Niering M. *et al.* Measurement of the hydrogen 1S-2S transition frequency by phase coherent comparison with a microwave cesium fountain clock. Phys. Rev. Lett. 84, 5496 (2000). 10990978 10.1103/PhysRevLett.84.5496

[b18] Rosenband T. *et al.* Frequency Ratio of Al^+^ and Hg^+^ Single-Ion Optical Clocks; Metrology at the 17th Decimal Place. Science 319, 1808–1812 (2008). 18323415 10.1126/science.1154622

[b19] Lämmerzahl C., Dittus H., Peters A. & Schiller S. OPTIS: a satellite-based test of special and general relativity. Classical and Quant. Grav. 18, 2499 (2001).

[b20] Reinhardt S. *et al.* Test of relativistic time dilation with fast optical atomic clocks at different velocities. Nature Phys. 3, 861–864 (2007).

[b21] Reigber C. *et al.* An Earth gravity field model complete to degree and order 150 from GRACE: EIGEN-GRACE02S. J. Geodyn. 39, 1–10 (2005).

[b22] Jentsch C. *et al.* HYPER: A satellite mission in fundamental physics based on high precision atom interferometry. Gen. Relat. Gravitat. 36, 2197–2221 (2004).

[b23] Fridlund C. V. M. Darwin-the infrared space interferometry mission. ESA bulletin 103, 20–25 (2000).

[b24] Lawson P. R. & Dooley J. A. Technology plan for the terrestrial planet finder interferometer. Publ. Jet Propulsion Laboratory 05-5, 1–149 (2005).

[b25] Fridlund M. Future space missions to search for terrestrial planets. Space Sci. Rev. 135, 355–369 (2008).

[b26] Baumann E. *et al.* High-performance, vibration-immune fiber-laser frequency comb. Opt. Lett. 34, 638–640 (2009). 19252577 10.1364/ol.34.000638

[b27] Sinclair L. C. *et al.* Operation of an optically coherent frequency comb outside the metrology lab. Opt. Express 22, 6996–7006 (2014). 24664048 10.1364/OE.22.006996

[b28] National aeronautics and space administration (NASA). Payload test requirements: NASA technical standard. https://standards.nasa.gov/documents/detail/3314910/, (2004) (Date of access: 01/02/2014).

[b29] Lezius M. *et al.* Radiation induced absorption in rare earth doped optical fibers. IEEE Trans. Nucl. Sci. 59, 425–433 (2012).

[b30] Fox B. P. *et al.* Effect of low-earth orbit space on radiation-induced absorption in rare-earth-doped optical fibers. J. Non-Cryst. Solids 378, 79–88 (2013).

[b31] Jung I. D. *et al.* Experimental verification of soliton mode locking using only a slow saturable absorber. Opt. Lett. 20, 1892–1894 (1995). 19862193 10.1364/ol.20.001892

[b32] National aeronautics and space administration (NASA). Combination methods for deriving structural design loads considering vibro-acoustic, etc., responses. http://www.nasa.gov/offices/oce/llis/0652.html, (1999) (Date of access: 01/02/2014).

[b33] Wertz J. R. & Larson W. J. Space Manufacture and Test. Space mission analysis and design. (ed. Wertz, J. R.) 519–532 (Microcosm Press, El Segundo, California, 1999).

[b34] United States Department of Defense. MIL-STD-810G: Environmental Engineering Considerations and Laboratory Tests. http://www.assistdocs.com/search/document_details.cfm?ident_number = 35978&, (2008) (Date of access: 01/02/2014).

[b35] Barth J. L. *et al.* Space, atmospheric, and terrestrial radiation environments. IEEE Trans. Nucl. Sci. 50, 466–482 (2003).

[b36] Girard S. *et al.* Proton- and gamma-induced effects on Erbium-doped optical fibers. IEEE Trans. Nucl. Sci. 54, 2426–2434 (2007).

[b37] Adams L. & Holmes-Siedle A. The development of an MOS dosimetry unit for use in space. IEEE Trans. Nucl. Sci. 25, 1607–1612 (1978).

[b38] The Space Environment Information System. http://www.spenvis.oma.be/, (2013) (Date of access: 01/02/2014).

[b39] Thomas J. *et al.* Radiation-resistant erbium-doped-nanoparticles optical fiber for space applications. Opt. Express 20, 2435–2444 (2012). 22330481 10.1364/OE.20.002435

[b40] Girard S. *et al.* Radiation-hard erbium optical fiber and fiber amplifier for both low- and high-dose space missions. Opt. Lett. 39, 2541–2544 (2014). 24784040 10.1364/OL.39.002541

