# Supplementary material for: Testing of a femtosecond pulse laser in outer space
Source: Sci Rep. 2014 May 30;4:5134. doi: 10.1038/srep05134 (PMC4038913; doi:10.1038/srep05134)
Supplement: Supplementary Information [file srep05134-s1.pdf]

Supplementary Information

Testing of a femtosecond pulse laser in outer space

Joohyung Lee, Keunwoo Lee, Yoon-Soo Jang, Heesuk Jang, Seongheum Han, Sang-Hyun Lee, Kyung-In Kang, Chul-Woo Lim, Young-Jin Kim, and Seung-Woo Kim**

1. Mechanical structure design and vibration analysis

The FSO payload was enclosed by a mechanical structure (black anodized 6061-T6 aluminium) designed to maximize the protection capability against vibration and heat within the given constraints on its volume, footprint and weight as shown in Fig. S1a. Vibration and heat transfer analysis was performed by using the Finite Element Method (FEM), of which the 3-dimensional grid model and material properties are described in Fig. S1b.

a

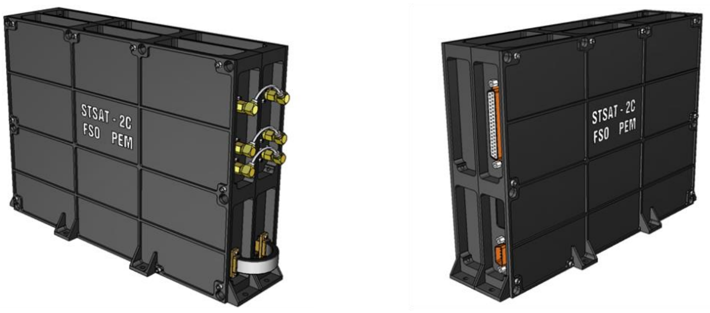

b

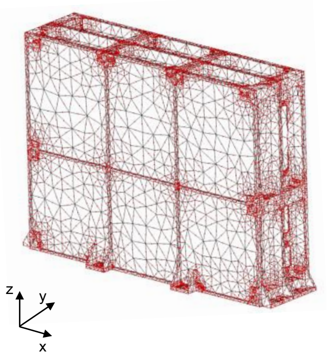

FEA analysis of FSO
(Black line : mesh, Red dot: node)

Item	Description
Software	CATIA V5R21
Element	parabolic tetrahedral
No. Node	291,418
No. Element	146,151
Mass	2.4 kg

Material	Density [kg/m³]	Elasticity [GPa]	Poisson	Strength [Mpa]	Remarks
Al6061-T6(1)	2,700	69	0.33	240 (Yield)	Box, Cover
FR4(2)	1,820	24	0.136	276 (Yield)	PCB
POM(3) (Derlin ®)	1,420	2.8	0.35	69 (Yield)	Fiber Housing

(1) MIL-HDBK-5H (2) Dupont, H-76836 (3) Plastic international

Figure S1. Mechanical structure of the FSO payload. a, Exterior views of mechanical structure and electrical interfaces (drawn by J.L). **b,** Finite element model constructed with tetrahedral meshes of 146,151 elements and 291,418 nodes.

FEM analysis was conducted to estimate the structural loading and natural frequencies during the launching stage. This analysis confirmed that the safety margin for structural failure exceeds 1.25 for metal parts and 2.00 for non-metal parts (Fig. S2a & S2b). In addition, the lowest natural frequency was calculated to be 204 Hz (Fig. S2c), which was found safe enough as it was 7.4 times higher than the satellite's lowest natural frequency of 27.5 Hz. This FEM analysis result was in good agreement with the actual vibration test result within a ~5.3 % discrepancy, assuring that the mechanical enclosure was robust enough to shelter the FSO payload.

a

Part Name	Material	Max. Stress (MPa)			Safety factor requirement	Criterion (σ_Y /S.F)	Margin of Safety	Compliance
		G_x	G_y	G_z				
Electronics Box	Al6061	29.82	114.59	26.40	1.25	192	0.68	Y
Laser Box	Al6061	28.80	103.08	34.60	1.25	192	0.86	Y
Front Cover	Al6061	28.64	142.27	43.02	1.25	192	0.35	Y
Back Cover	Al6061	26.50	142.45	22.88	1.25	192	0.35	Y
Fiber Housing Plate	POM	8.78	10.01	9.27	2	34.5	2.45	Y
Fiber Housing Cover	POM	2.54	4.88	3.65	2	34.5	6.07	Y
Main PCB	FP4	15.25	25.00	13.72	2	138	4.52	Y
Laser PCB	FP4	5.14	32.29	10.21	2	138	3.27	Y

b

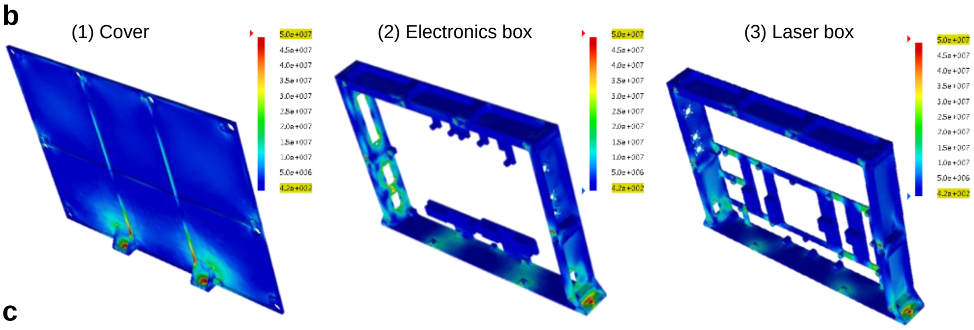

c

Mode	Freq. (Hz)	T_x (%)	T_y (%)	T_z (%)	R_x (%)	R_y (%)	R_z (%)
**1**	**204**	**0.00**	**45.80**	**0.09**	**0.00**	**2.94**	**0.00**
2	225	0.00	1.89	0.00	0.00	2.82	0.00
3	247	0.00	10.98	0.02	0.00	2.20	0.00
4	284	0.00	5.53	0.20	0.00	3.23	0.00
5	320	0.00	0.14	0.12	0.00	0.03	0.00
6	331	0.00	0.46	0.03	0.00	0.70	0.00
7	403	0.00	1.59	0.01	0.00	2.67	0.00
8	435	0.01	0.02	0.00	0.00	0.00	1.53
9	475	0.00	0.00	0.01	0.00	0.00	0.42
10	482	0.00	0.09	0.00	0.00	0.02	0.46
	**Total**	**0.01**	**66.51**	**0.47**	**0.00**	**14.63**	**2.41**

Figure S2. FEM analysis. a, Structural stress analysis under a steady-state acceleration of 490 m/s^2 (50 g). **b,** Von-Mises stress distribution in the cover plate (1), electronics box (2) and laser box (3). **c,** Vibrational mode analysis results show the two lowest resonance natural frequencies located at 204 and 225 Hz. T_x, T_y and T_z (R_x, R_y and R_z) indicate the translational (rotational) inertia along (around) x, y and z directions at each vibrational mode.

2. Electronics design for remote operating and monitoring

The electronic unit (Fig. S3) was configured for remote operation and monitoring of the FSO payload; temperature and current control of pump laser diodes (LDs), data acquisition from photo-detectors (PDs), data storage to static random-access memories (SRAMs), communications with the mother satellite and power supply. All the functions are made to be supervised by two main processors: a micro-controller unit (MCU) (C8051, Silicon Laboratories) and a field-programmable gate array (FPGA) (Actel Axcelerator, A3P600). The MCU is preprogramed to deal with all the commands sent from the satellite on-board computer (OBC), while the FPGA is in charge of data storage to four 1024 kB SRAMs (CY7C10612, Cypress) and data-transfer to the main memory of the mother satellite. An oven-controlled crystal oscillator (OCXO) supplies reference clock signals to the MCU, FPGA and a high-speed analogue-to-digital converter (ADC). This clock signal is also used as the reference frequency signal for super-heterodyning RF signals for frequency counting or phase-locking control.

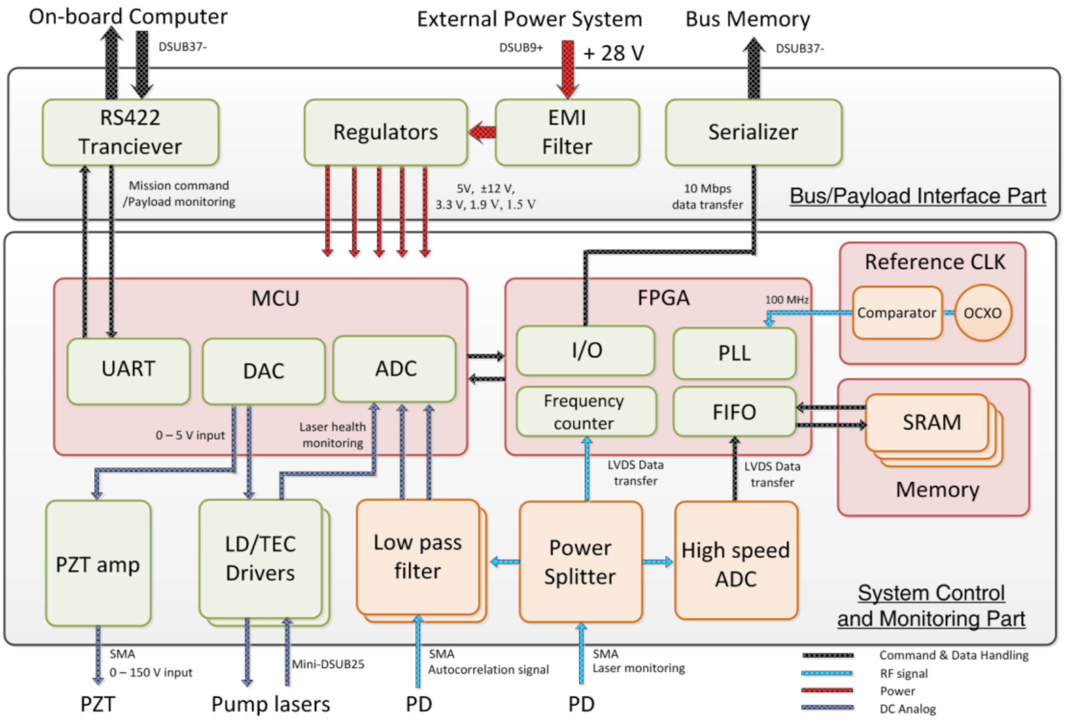

Figure S3. Functional block diagram of FSO's electronic unit. EMI: electromagnetic interference, MCU: micro-controller unit, UART: universal asynchronous receiver/ transmitter, DAC: digital-to-analogue converter, ADC: analogue-to-digital converter, FPGA: field programmable gate array, I/O: input/output, PLL: phase-locked loop, FIFO: first in first out, CLK: clock, OCXO: oven-controlled crystal oscillator, SRAM: static random-access memory, LD: laser diode, TEC: thermoelectric cooler, PD: photo diode.

The pump LDs and PZT actuator are controlled by the MCU through a digital-to-analogue converter (DAC), a high-voltage PZT amplifier (PZM-B, Matsusada) and two electronic chips for temperature control (TEC, WTC3243, Wavelength Electronics) and

current control (FL500, Wavelength Electronics). The slow signals such as the average power, autocorrelation, LD temperature, and LD injection current are detected and recorded directly by the MCU. At the same time, the FPGA is set to run at a 200 MHz sampling rate so that the fast RF pulse train signal is counted through frequency down-conversion to 200 kHz by super-heterodyning with the reference clock signal supplied from the OCXO.

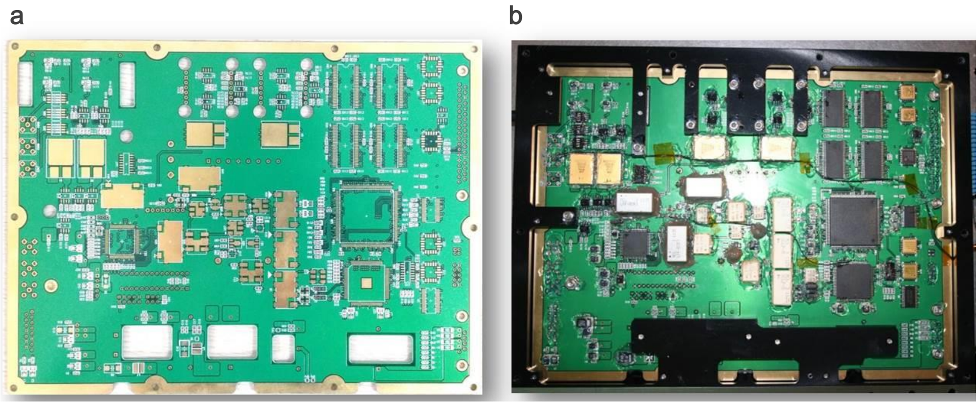

Figure S4. FSO electronics. a, Printed circuit board (PCB) layout design. **b,** Electronic board after assembly. Heat generating electrical components were connected to the metal pad of the enclosing structure for dissipation though conduction.

3. Unbalanced interferometer for pulse characterization

For monitoring of the output pulse duration as well as optical spectrum during operation in orbit, the FSO payload was equipped with an unbalanced Michelson interferometer (Fig. S5a) specially designed to generate the 1st order cross-correlation signal based on the principle of optical sampling by cavity tuning [S1-S4]. (The temporal time delay Δt between the two pulses recombined at the 5:5 coupler is expressed as $\Delta t = m \cdot c \cdot f_r / 2f_r^2$ in which c is the velocity of light in the fiber medium, f_r is the pulse repetition rate and m represents the positive integer proportional to the optical path length difference between the interferometer arms.) The time delay Δt was made to scan from 0 to 18 ps as the pulse repetition rate f_r was varied by the on-board PZT actuator over a tuning range of ±0.4 kHz around 25 MHz. Exemplary measurements of the pulse duration (by 1st order cross-correlation) and its corresponding optical spectrum (by Fourier-transform of 1st order cross-correlation) are shown in Fig. S5a and S5b.

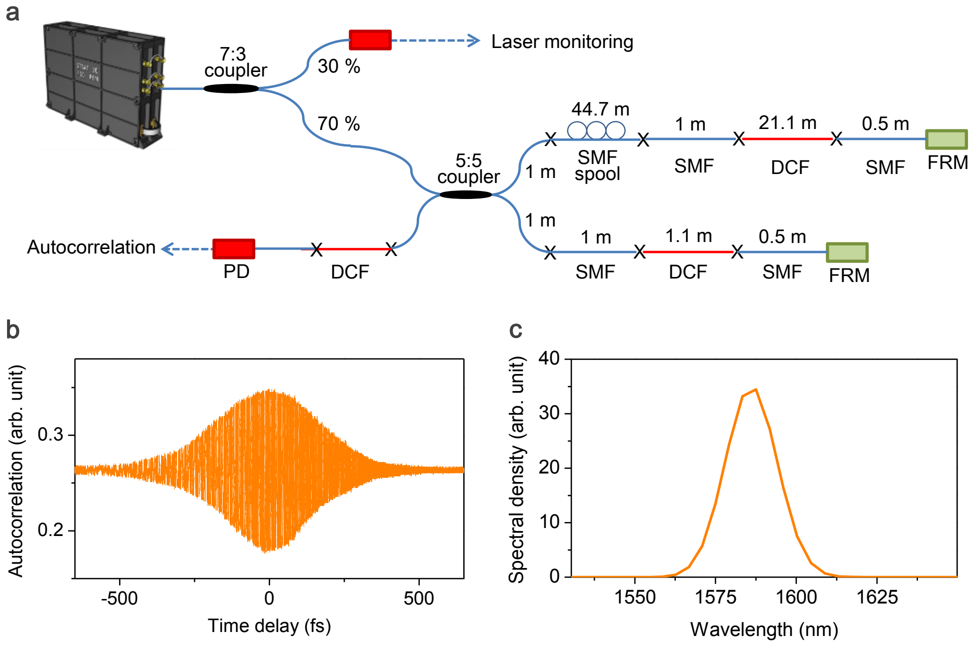

Figure S5. Unbalanced fiber interferometer for pulse characterization. a, Design layout of the unbalanced interferometer. **b**, First-order cross-correlation to monitor the pulse duration. **c,** Optical spectrum obtained by Fourier-transform of the first-order cross-correlation signal. SMF: single mode fiber, DCF: dispersion compensating fiber, FRM: Faraday rotating mirror.

4. FSO environmental tests on the ground

A. Vibration test

For vibrational test, three acceleration sensors were installed in x-, y- and z- direction on the mechanical enclosure of the FSO payload (Fig. S6). An electro-magnetic vibration machine (LING, 1216VH) was used to generate random vibration with a trapezoidal acceleration profile for 60 seconds; the integrated acceleration was set at 139 m/s^2 (14.2 g) as required. Acceleration was measured repeatedly for each vibration axis; the resonance frequencies along the x-direction were measured to be 193 and 220 Hz. This result agreed with the lowest natural frequencies predicted by the FEM analysis within 5.3 deviation. The measured natural frequencies were far beyond those of the mother satellite - 27.5, 27.5 and 65.8 Hz along the x-, y- and z-direction, respectively. It was noted that natural frequencies measured at several separate tests remained unchanged, indicating there was not significant structural damage incurred during the whole process of vibration test.

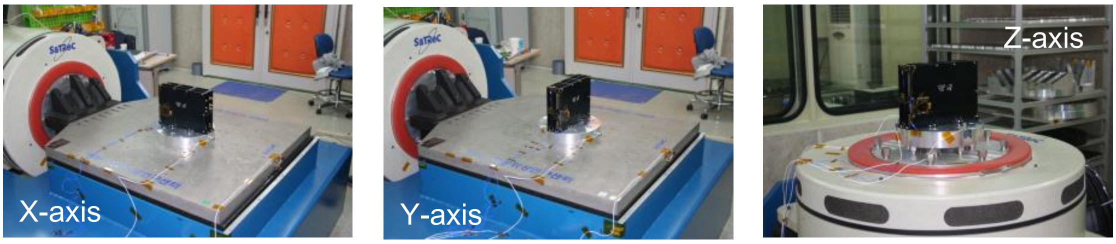

Figure S6. Vibration test setup using three accelerometers on an electro-magnetic exciter generating random vibration.

B. Thermal vacuum test

A thermal-vacuum chamber (Thermotron) was used for temperature control from -10 to 50 ^{o}C under a vacuum of 10^{-3}~10^{-4} Pa (10^{-5}~10^{-6} torr). The temperature profile specified in Fig. 3b was provided for 2.5 operational test cycles and 8 survival test cycles with a 90 minute dwell time at each temperature peak and valleys During the operational cycles, the mode-locking state of the FSO payload was monitored while the chamber temperature was varied between 0 to 45 ^{o}C. The pump LD temperature was also observed simultaneously so that a feasible zone for normal operation was established (Fig. S7). In the survival test, the operation state of the FSO payload was verified before and after the full test cycles were completed.

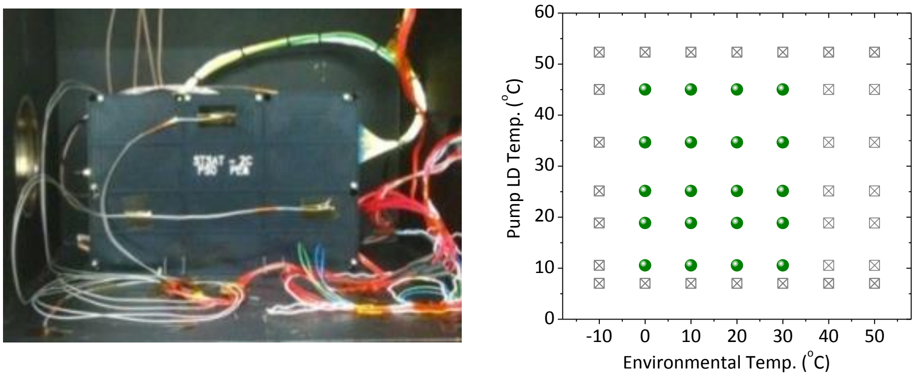

Figure S7. Thermal vacuum test. The feasible combination of the environment temperature and pump LD temperature was identified (left) for normal operation of the FSO payload in space.

C. Gamma-ray radiation test

The FSO payload assembly was exposed to ^{60}Co gamma-ray radiation (Fig. S8). An accumulated amount of 147 krad total ionizing dose (TID) was irradiated over 17 hours with an 8.73 krad/hour exposure rate while temperature was kept constant at ~15 ^{o}C. As explained in the main text and Fig. 3c, the FSO payload began to break down when the gamma-ray TID reached 31.6 krad. After the radiation test, the FSO payload was fully recovered to normal operation by simply replacing the radiation-exposed Er-doped fiber with a new piece of fiber, confirming that the Er-doped fiber was only the component damaged during the radiation test.

Individual tests were also made on the saturable absorber (SA), photodiodes (PDs) and pump laser diodes (LDs) used as key components in the FSO payload since high-energy radiation may induce unexpected deterioration in the energy band gap of semiconductor devices [S5,S6]. Total ionization dose (TID) over 200 krad – which corresponds to a 45 year operation in the low earth orbit of the FSO payload with a 1.6 mm-thick aluminium shield – was irradiated on each component under test. The PDs operation was monitored while a constant optical power of 10 mW was illuminated on it using a distributed feedback laser which was radiation-protected by a leaden shield block. The monitored PDs output voltage decreased by 0.12 % at a 4.7 krad TID which corresponds to a one year operation in outer space (Fig. S8b). For the LDs test, a photo-detector in a leaden radiation-shield was used to monitor the LD output power variation which showed a 0.23 % decrease during 4.7 krad TID exposure (Fig. S8c). After a 220 krad TID radiation, the LDs output power was measured by varying the injection current (Fig. S8d): the sensitivity slope decreased from 0.76 to 0.73 mW/mA by 3.9 %. Finally, for the SA test, the saturation fluence, the modulation depth and the fast/slow recovery time were measured using a pump-probe test setup under gamma-ray radiation; there was no noticeable change was observed over ~50 krad. Detailed discussion on space radiation effects of the SA was reported in a separate paper [S7]. All these individual tests conclusively confirmed that the SA, PDs and LDs were more robust to space radiation than the Er-doped gain fiber.

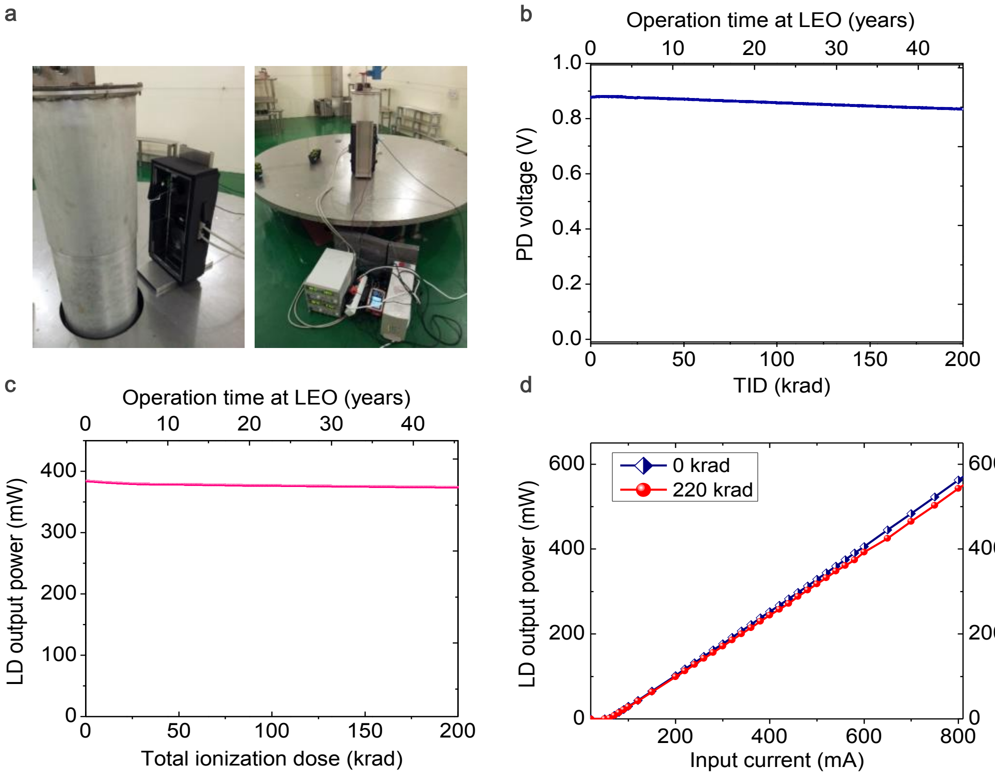

Figure S8. Space radiation tests. a, Gamma-ray radiation test setup. **b,** PDs sensitivity variation under gamma-ray radiation. **c,** Pump LDs power change under gamma-ray radiation. **d,** LDs current-power slope change before and after 220 krad TID gamma-ray exposure.

5. System assembly and integration

The flying model of the FSO payload was assembled with all fiber-optics being fusion-spliced with connection loss less than 0.1 dB, hermetically sealed with low-volatility silicon and then packaged into a polyoxymethylene frame of low thermal conductivity (Fig. S9). Then the electric signal lines to the LDs, PDs and PZT were wired through coaxial connectors within the electronic compartment. The FSO payload was finally installed on the second floor of the Naro science satellite which was later moved to the launching station, the Naro Space Center, located in Goheung, South Korea by a special anti-vibration truck.

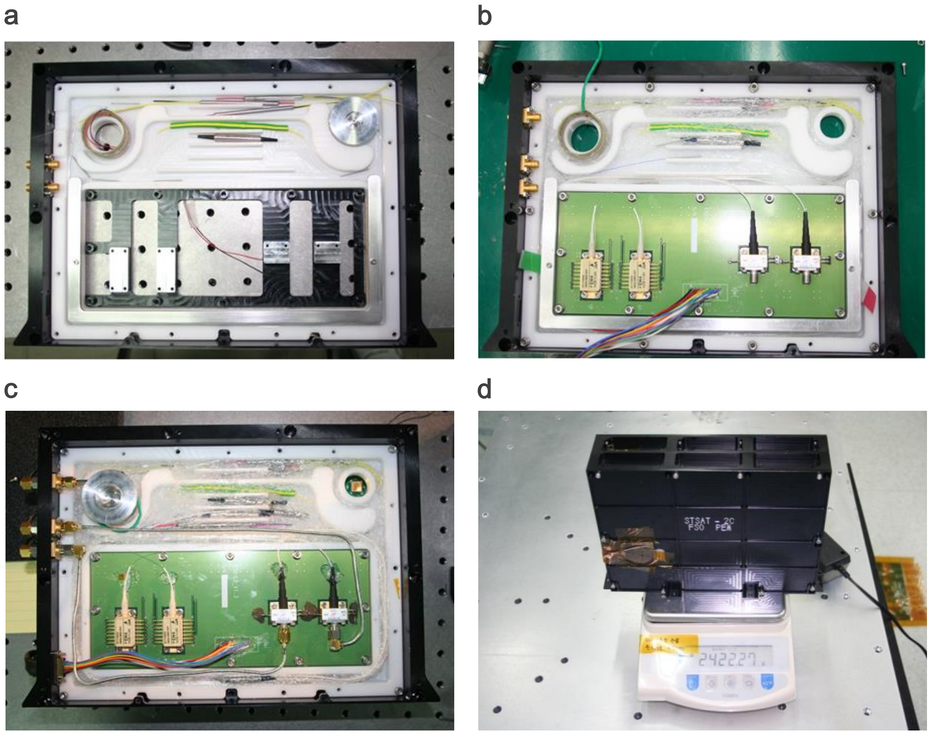

Figure S9. The FSO payload assembly. a, Installation of fiber-optic components **b,** Fiber sealing with space-grade silicon. **c,** Electric signal connection lines. **d,** Exterior view of the FSO payload finally assembled.

References in supplementary information

S1. Wertz, J. R. & Larson, W. J. *Space mission analysis and design* Edn. 3 (eds Larson, W., & Wertz, J.) 519-532 (1999).

S2. Joo, W.-D., *et al.* “Femtosecond laser pulses for fast 3-D surface profilometry of microelectronic step-structures,” *Opt. Express* **21,** 15323-15334 (2013).

S3. Potvin, S., Boudreau, S., Deschenes, J.-D., & Genest, J., “Fully referenced single-comb interferometry using optical sampling by laser-cavity tuning,” *Appl. Opt.* **52,** 248-255 (2013).

S4. Hochrein, T., *et al.* “Optical sampling by laser cavity tuning,” *Opt. Express* **18,** 1613-1617 (2010).

S5. Johnston, A.H. *et al.* Radiation degradation mechanisms in laser diodes. *IEEE Trans. Nucl. Sci.* **51,** 3564-3571 (2004).

S6. Uffelen, M.V. *et al.* Wavelength dependence of the response of Si and InGaAs pin photodiodes under gamma radiation. *Proc. of SPIE* **5554,** 132-143 (2004).

S7. Jang,Y.-S., *et al.* Space radiation test of saturable absorber for femtosecond laser. *Opt. Lett.* **39** (Early Posting).
